# “The potential of social media in health promotion beyond creating awareness: an integrative review”

**DOI:** 10.1186/s12889-022-14885-0

**Published:** 2022-12-21

**Authors:** Atousa Ghahramani, Maximilian de Courten, Maria Prokofieva

**Affiliations:** 1 Victoria University, Business School, 300 Flinders St, Melbourne, VIC 3000 Australia; 2 Victoria University, Mitchell Institute for Education and Health Policy, 300 Queen St, Melbourne, VIC 3000 Australia

**Keywords:** Social media, Health promotion, Health awareness, Health behaviour

## Abstract

**Background:**

Developing strategies to change health behaviour is one of the biggest challenges of health promotion programs. Social media, as a popular and innovative communication and education tool, offers opportunities to modify health behaviour. While literature on using social media for health promotion campaigns is growing, there is a need to evaluate the approaches used to change health behaviour, rather than only creating awareness.

**Objective:**

The paper reviewed the literature on application of social media in health promotion campaigns with a particular focus on the methodologies used in assessing the outcome of the programs for behaviour change. This fills the void in collating evidence to extend health promotion campaigns to effect sustainable behavioural change.

**Method:**

Peer-reviewed articles were identified through multiple science databases. A systematic electronic search was conducted to retrieve review and original papers published between January 2010 and April 2022. The titles and abstracts of the articles were screened according to inclusion and exclusion criteria. All authors independently read the full texts and discussed them to reach a consensus about the themes. Concept mapping was used to present results from analysis of the included papers.

**Results:**

Of the 674 citations, 28 (4.1%) studies were included in this review. The methodology approaches of 18 (2.7%) papers, that aimed to evaluate the impact of social media in health promotion campaigns towards behaviour change, were analysed further using concept mapping. The results showed that 10 studies (55.5%) adopted quantitative methods and five studies (27.7%) used mixed methods and three studies (16.6%) used qualitative methods. Facebook and YouTube were used more for intervention purposes to change health behaviour. Twitter and Instagram were used more to observe the trend of changes in health behaviour. Six studies (33.3%) adopted Social Cognitive Theory and one study (5.5%) applied the Transtheoretical Model as the framework to evaluate the outcome. Overall, the results show that though social media has potential in promoting behaviour change, the estimation of this change in long-term lies outside the scope of social media health campaigns. This is also reflected in the methodologies used in existing studies to assess such sustainable changes. The employed measures usually target immediate behaviour or social media engagement rather than addressing the change on a behavioural level.

**Conclusion:**

Evaluating the performance of social media campaigns to promote health behaviours towards a sustainable outcome is a complex process. Emerging research is focused on evaluating the potential of social media as an opportunity to create awareness. Such measures require less effort in quantifying and isolating the effect. The design of the campaigns is required to be aligned in relation to stages of the behaviour change. The study provides suggestions on how this can be achieved.

## Background

The review of existing literature in application of social media in health promotion campaigns revealed a range of negatives and positives aspects of using this venue for public health and medicine and challenged its effectiveness to ensure behaviour change, especially in the long-term perspective. While some positive aspects include potential access to wider target audience, more accurate health messaging and facilitated communication between health professionals and public, the effectiveness of such health promotion campaigns and their evaluation are somewhat questioned by improper communication strategies, lack of support, self-diagnostic and concerns about short- and long-term changes in health behaviour.

However, clearly better research designs are needed to measure the effectiveness of social technologies [1].

Social media serves as a collaborative dissemination platform to reach and influence the target audience and deliver health related information [2]. Social media can provide efficient, ubiquitous and user-friendly approaches to attract large numbers of participants and demonstrate a certain level of engagement with the health-related messages [3]. Social media interventions may improve early diagnosis of diseases and facilitate behaviour change techniques, such as providing social support and emphasising the consequences of a health issue [4]. Research has revealed a significant positive influence of social media on public health protection [5] with the potential to change individuals’ behaviour towards establishing a healthy lifestyle [6–8].

Preliminary evidence shows that social media interventions can effectively promote health behaviour change [9]. Health promotion campaigns effectively contribute to increased awareness of health issues using social media to enhance the willingness of social media users to discuss the issues openly [5]. Recent events related to COVID-19 are a vivid example where social media was used to significantly raise awareness of public health topics and elicit behavioural changes of individuals and thus increased protection against COVID-19 [5]. However, there are challenges in evaluating the impact of social media interventions in health promotion programs and understanding how desired outcomes could be achieved for sustained engagement and behaviour changes [4, 9]. The majority of past studies analysed social media from the viewpoint of the organisations implementing health promotion campaigns and only limited studies evaluated short-term and long-term changes in individuals’ health behaviour and lifestyle and validated the effectiveness of social media beyond creating awareness [5, 10]. While social media campaigns can provide a significant change in health behaviour and orchestrate health promotion efforts [11], the evaluation methods to measure the outcome and analyse the success of health promotion programs using social media platforms remain unexplored [12].

Use of social media in health promotion campaigns are of high interest among health professionals, marketers, policy setters, regulators, and any health enthusiasts. But evaluating the impact of such is a complex process [2]. The main reason is that these initiatives are not implemented in isolation, but rather complementary to each other, creating synergies and blurring the effect. Such synergies make it difficult to isolate the impact of individual social media in health promotion campaigns, as well as potentially, make the impact marginal or complementary to other factors. For example, obesity and diabetes control involves individual knowledge, awareness and motivation for both diet and exercise, but it also involves structural and contextual factors such as urban design, food availability and pricing, access to appropriate clinical services etc. [13]. Similarly, smoking cessation involves taxation, restrictions on how and where tobacco products are marketed and sold, smoke free public spaces etc. [14]. A systematic review found that using online social networks may not enhance smoking cessation or weight loss [15]. However, some studies concluded that use of social media can improve physical activity behaviours [16] or facilitate healthier choices of eating and recipe tips in young adults, although they are reluctant to share their information on the online social networks [17].

The current study developed a framework to guide the assessment of social media and illustrate the potential functions of social media in promotion of public health. The examples of functions that can influence the outcomes relevant to the evaluation of the effects of social media on the public health are minimising the spread of misinformation and increasing public awareness of accurate health-related information, enhancing real-time surveillance related to incident disease, disease Control and mitigation, and screening or treatment interventions [18].

This paper develops a multifaceted review to address this gap and extends the study to provide a structured and comprehensive map of methods used to evaluate the impact of social media beyond awareness and on health behaviour change. The specific research questions were: (1) What is the potential of social media in health promotion beyond creating awareness to promote health behaviour change? (2) What methodologies have been used to assess the impact of social media on health behaviour change?

## Methods

### Overview

Integrative reviews are a unique approach allowing for the inclusion of diverse methodologies and can be used to analyse literature providing a more comprehensive understanding of a phenomenon that existed prior to the review [19]. It combines data from various research designs including experimental and non-experimental research [20]. The integrative method allowed a comprehensive understanding of the gaps in the field along the stages of a) literature search, b) data collection, c) data analysis and d) presentation of the results [20].

### Search strategy

We conducted a systematic electronic search on Sage, PubMed, Web of Science and Scopus databases. The used search string was “social media” AND “health promotion” AND (“campaign” OR “social marketing”) AND “health behaviour change”. However, there were concerns that we missed research studies. Therefore, this study was supplemented by simply implementing a search on Google Scholar, as the source of scientific papers, using the same keywords; “social media” AND “health promotion” AND (“campaign” OR “social marketing”) AND “health behaviour change”.

### Inclusion and exclusion criteria

The inclusion criteria were: (a) published in English language; (b) published in peer-reviewed journals; (c) review papers and original research studies published between January 2010 and January 2022; (d) all study designs. The exclusion criteria were: (a) published editorials, opinions, discussions, dissertation thesis, reports, conference papers and abstracts; (b) studies with a primary focus on marketing or advertising, using social media with digital media (i.e. apps, e-health) or mass media (i.e. TV, Radio, newspaper), context and social network analysis. Criterion (c) was informed by the preliminary research on the timing of the development of social media platforms, including the year of launch and development of critical mass of social media users active on the platform [21, 22]. Duplicate papers were removed, and one researcher (AGh) read the titles and abstracts to select the most relevant studies.

The review was conducted as the integrative review, which allowed inclusion of both experimental and non-experimental studies. The quality of the publications was assessed in accordance with PRISMA2020 guidelines [23]. The quality assessment and risk bias in the studies were addressed by reviewing the study design of the selected papers regarding the goals of social media campaigns, social media platforms used/reviewed in the study, participants characteristics and communication of results. Attention was given to ensure relevance, reliability and comparability of the results. External indicators, such as citation index of the included papers, rankings of the publication venue and time/relevance of the publication, were considered.

### Screening and selection of papers

Study selection is typically a multi-stage process in which potentially eligible studies are first identified from screening titles and abstracts, then assessed through full text review and, where necessary, contact with study investigators. Increasingly, a mix of screening approaches might be applied (such as automation to eliminate records before screening or prioritise records during screening). In addition to automation, authors increasingly have access to screening decisions that are made [23]. The titles and abstracts of the papers were screened for the relevance and the selection criteria. The identified papers from the searches were screened by two authors (AGh and MP), who first independently screened the search results and agreed on inclusion. For full text screening, three authors (AGh, MP, MDC) independently screened the articles and agreed on 90% of the articles. One of the researchers (AGh) reviewed the studies and selected the papers with the inclusion criteria to address the identified research questions. One researcher (AGh) started to construct the concept maps. The two other researchers (MP and MDC) verified the process of constructing concept mapping and critically evaluated the produced concept maps. One researcher (AGh) added the selected examples to the main concepts. Three researchers (AGh, MP, MDC) independently reviewed and evaluated the studies and reached consensus on the inclusion for data analysis. An agreement was indicated between the researchers and the discrepancies were discussed with reference until consensus was achieved.

### Presentation and synthesis of the results

The main difference between an integrative review and a systematic review is the types of studies that are included in the review. Systematic reviews include experimental studies, and many times only randomised controlled trials. Integrative reviews include both experimental and non-experimental studies. While the scoping review aims to map the literature, seeking to describe the results in a graphic and classificatory way to have a better idea on what is there, Integrative review proposes to integrate the literature found on a determined object of investigation.

This paper aims to present a comprehensive overview and assessment of the main approaches and describe various approaches by combining, integrating, and synthesising research findings. We conducted an integrative review of the literature to explore the historical, contextual, and evolving nature of research synthesis [24]. The synthesis of the results was done by triangulation of qualitative and quantitative synthesis approaches, while the qualitative content analysis was the main method employed in the study. This paper presents a new strategy for reviewing multidisciplinary academic literature [25]. Constrained and unconstrained computer assisted text analysis was explored using available R package functionalities (e.g revtools, statcheck, metafor packages).

## Result

### Overview of the process of paper selection

**Fig. 1:**
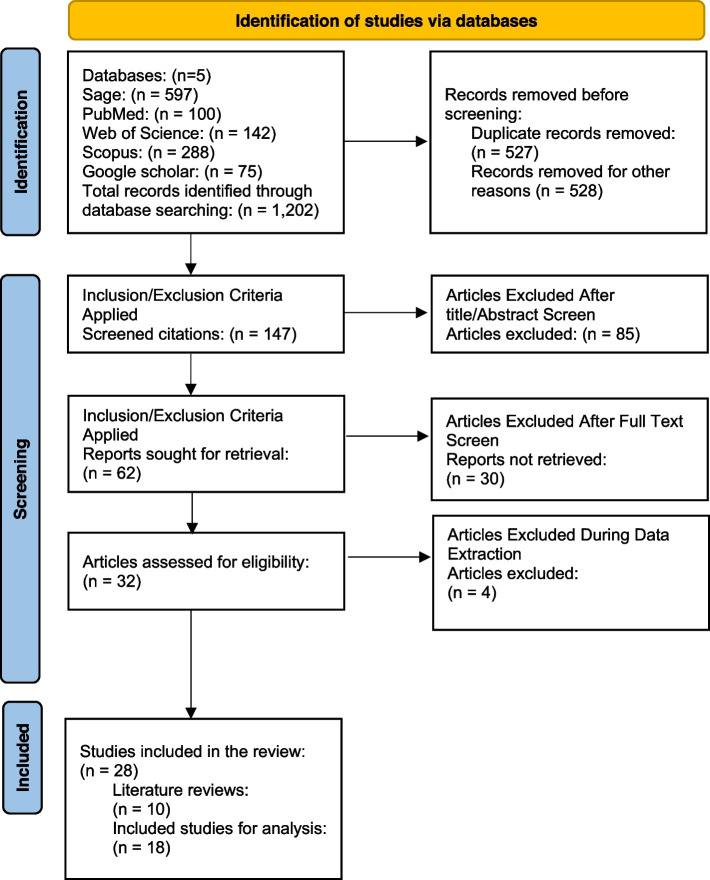
Flowchart of the selection process of the studies based on PRISMA2020 guideline

After removing duplicated papers and excluding ineligible articles, 28 studies met the inclusion criteria and 18 original research papers were analysed further to examine the methodology approaches to evaluate the outcome of health promotion campaigns for health behaviour change. The flowchart (Fig. 1) details the process of identification and selection of the research papers based on the PRISMA2020 guideline.

### Overview of included studies in application of social media to promote health behaviour

Of 28 included studies, 10 studies were review papers including five literature reviews [4, 9, 11, 26, 27], four systematic reviews [5, 16, 17, 28] and one scoping review [4]. Eighteen research papers evaluated the outcome of health promotion campaigns using social media to promote health behaviour change [5, 10, 12, 29–43]. The potential of social media platforms was explored for a range of health behaviour and prevention topics including overweight and obesity [34, 42], smoking cessation [31, 32, 36, 40], cancer prevention [12, 30], diabetes [29], oral health [35], eating disorder [37], COVID-19 prevention [5, 10], suicide prevention [33, 38] and mental health and wellbeing [39]. We summarised the included studies (Table 1) based on objective, study design, used social media platforms, used theoretical framework and the expected health behaviour outcome.

**Table 1:** Summary of the included papers evaluating social media use for health behaviour outcome

Study	Objective	Study design	Social media	Theory framework	Behavioural outcome
Merchant et al. [34]	Examine participant engagement	Mixed method Content analysis Semi-structured interview	Facebook	SCT**	Overweight/obesity control
Thrul et al. [36]	Increasing engagement	Quantitative Content analysis Interventional	Facebook	TTM*	Smoking cessation/prevention
Jiang and Beaudoin [32]	Increasing online engagement	Quantitative Content analysis Descriptive analysis	Twitter	SCT**	Smoking cessation/prevention
Gough et al. [30]	Examine the feasibility of social media to improved knowledge and attitudes	Mixed method Interventional Quasi-experimental Online survey Focus group interview Descriptive analysis	Twitter	No	Skin cancer prevention Improved attitudes toward UV exposure
Gabarron et al. [29]	Promote healthy lifestyles encourage engagement	Quantitative Content analysis Online survey Sentiment analysis Content analysis	Twitter Facebook Instagram	LM***	Diabetes prevention Communication behaviour
Yoo et al. [12]	Examine and predict the impact of communicative behaviour	Quantitative Observational Online survey Feasibility study	Twitter	SCT**	Cervical cancer prevention
Potts and Radford [35]	Examine geographical reach and engagement Oral health promotion	Mixed method Observational Content analysis Cross-sectional	Twitter	No	Oral health prevention
Viguria et al. [37]	Increasing engagement Increasing communication related to eating disorders	Mixed method Content analysis Observational Descriptive analysis	Twitter	No	Eating disorder prevention Help-seeking Treatment-seeking
Al-Dmour et al. [5]	Public health awareness to control pandemic	Quantitative Online survey Descriptive analysis	Various	SCT*	Covid 19 prevention
Hefler et al. [31]	Increasing online engagement	Quantitative Content analysis	Facebook	No	Smoking cessation/prevention
Okpara et al. [10]	Examining the impact of colour cartoons and predict recall	Quantitative Online survey Regression analysis	YouTube	SCT*	Covid 19 prevention
Cote et al. [38]	Improving mental health literacy Decreasing stigma Generating public discussion	Mixed method Content analysis Observational	Twitter	No	Suicide prevention
Loss and von Uslar [33]	Explore a range of prevention topics in the communications raised engagement	Quantitative Observational Content analysis Cross-sectional Non-experimental	Facebook	No	Increase health literacy
Dodemaide et al. [39]	phenomenological understanding of social media use for young adults	Qualitative Content analysis Cross-sectional online survey	Facebook/Twitter	No	therapeutic affordances Improve quality of life
Hefler et al. [40]	enhance Indigenous tobacco control	Qualitative Interview Content analysis	Facebook	SCT	smoking prevention
Kite et al. [41]	maximises user engagement	Quantitative Content analysis	Facebook	No	enhance communication
Naslund et al. [42]	social support for adopting healthier behaviours	Mixed method Feasibility study	Facebook	No	weight control for adults With serious mental illness
Sendall et al. [43]	engage this “hard-to-reach” groups	Qualitative interview Online survey	Facebook	No	reduce the risk of chronic disease

* *TTM* Transtheoretical Model

** *SCT* Social Cognitive Theory

*** *LM* Laugh Model

## Result presentation by concept map

This review adopted the concept map method based on the recommendation of Novak and Gowin [44] for both analysing the data and presenting the results. All three researchers (AGh, MP, MDC) were involved in creating the concept maps. The process of concept mapping are; (1) Identify a research question addressing a problem, issue or gap in knowledge, (2) understand the concepts, (3) create the concept map by placing the key concepts at the top of the hierarchy and select and arrange the defined concepts under the key concepts, (4) categorise the concepts in different domains, (5) a specific examples of events or objectives that clarify the meaning of the concept.

**Fig. 2:**
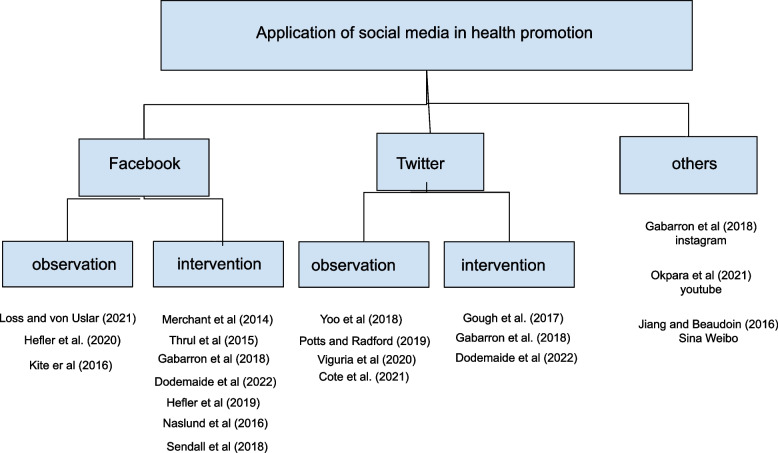
application of social media platforms to change health behaviour in the studies

The first constructed concept map (Fig. 2) adopts observation and intervention *as* the two main applications of social media in health promotion research [9]. It illustrates that Facebook was used more for intervention [29, 34, 36, 39] than observation [31, 33] purposes and Twitter was used more for observation [12, 35, 37, 38] than intervention [29, 30, 39] purposes. Sina Weibo [32] and Instagram were used for observation [29] and YouTube was used for intervention purposes [10].

### Application of Facebook

Existing research recognised Facebook as a social media platform used to change health behaviour [9, 39] with the potential to deliver strategies in successful health promotion interventions [34] to promote healthy lifestyles [29]. One study evaluated Facebook posts beyond reach, shares, and likes to identify important components that influence health behaviour [31]. Analysing the content of Facebook accounts of health insurance companies that promote health through education showed that many providers could not actively interact with a wider audience on Facebook [33]. One study investigated the impact of social media to reduce smoking habits and promote healthy behaviour among Indigenous people in Australia by sharing tobacco control content on Facebook [40]. One study explored the use of Facebook by Australian public health organisations to understand what features and strategies are associated with wider and bigger user engagement [41].

### Application of twitter

Twitter is applied as a supplementary tool to enhance social engagement by spreading information and strengthening social networks in various online health promotion programs as a potential source of public and searchable data relating to health behaviour [9]. One study tested the feasibility of designing, implementing, and evaluating social media interventions using Twitter data for prevention purposes [30]. Some studies [35, 37] investigated Twitter data to conclude how the majority of tweets can promote specific preventive behaviours. One study [38] explored the association between the content of tweets related to national mental health campaign and the real number of suicides to compare the suicide counts during the event to a control timeframe and the associated behaviour change to diminish suicide rates to promote help-seeking and resilience.

### Application of health promotion theory frameworks

It is important to observe the shifts in the stages of behaviour change of TTM to evaluate the sustained improvement of health behaviour outcomes that can be prescribed to individuals who are willing to quit an unhealthy habit and modify their behaviours [26]. Social media interactions provide rich data sources to understand the processes of stages of behaviour change and can help to discover the patterns of social behaviour changes [26]. Public health awareness requires the incorporation of some theories of behavioural change into social media health interventions [5].

The Transtheoretical Model (TTM) has been used in one study to conceptualise the process of behaviour change [36]. One study [36] identified the highest engagement in pre-action of stages of TTM behaviour change (Precontemplation, Contemplation, and Preparation) in a content intervention on Facebook.

**Fig. 3:**
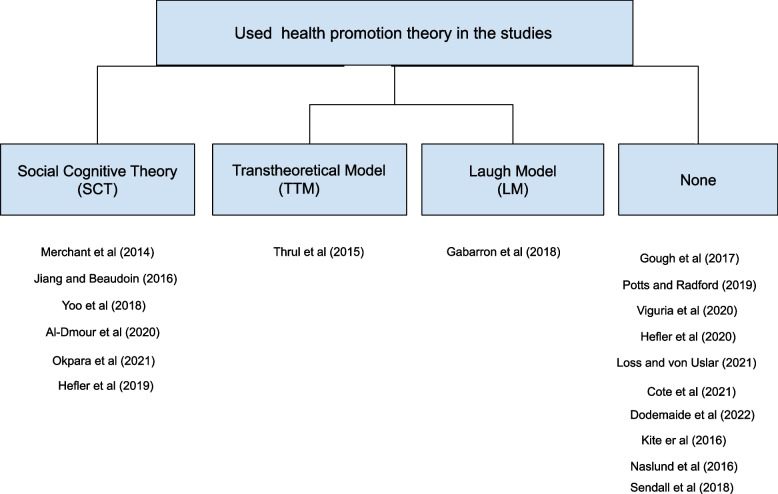
Health promotion theory frameworks used in the studies

Figure 3 illustrates that the majority of studies [5, 10, 12, 32, 34, 40] adopted social cognitive theory (SCT) as the basis to analyse the social media content and evaluate the relationship between the outcome and self-efficacy postings that influenced online audience engagement. Social cognitive theory (SCT) was used with the assumption that people adopt new behaviour better when they observe the others’ behaviour and demonstrate the desired health behaviour for social media users to learn through observation instead of other health messages like written texts or still pictures [10].

### The adopted methodological approaches in the studies

Existing research discovered that evaluation frameworks for social media interventions can help to measure behaviour change and found exposure, reach, and low- to medium-level of users’ engagement as the most important factors to be measured in the social media campaigns in health promotion [4].

**Fig. 4:**
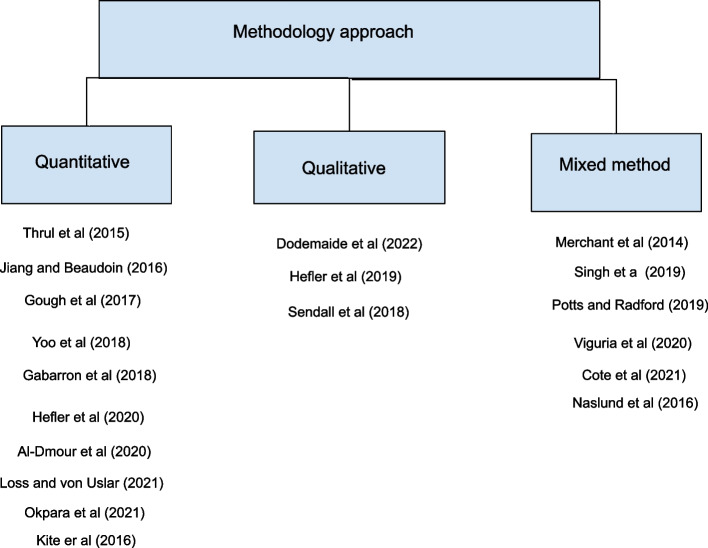
methodology approaches used in the studies

The results of concept map in Fig. 4 shows that the majority of the articles (65%) adopted quantitative approaches [5, 10, 28–33, 36] to evaluate the impact of social media in health behaviour change and 5 studies (35%) used a mixed method [3, 26, 34, 35, 37] for the study design to evaluate the outcome.

### The study design used in the original papers

While social media are becoming preferred methods of health promotion for showing their effectiveness in reaching public audiences [27], the area of need is detected in research to develop models that go beyond calculating reach, shares, and likes and evaluate the outcome of social media campaigns for health promotion [4, 31]. Most research addresses specific interventions and approaches, which vary widely in functionality, and usability. Health promotion evaluations typically seek to understand how effective these approaches are as tools for health promotion and public health communication, education, and behaviour outcomes related to the intervention [27].

**Fig. 5:**
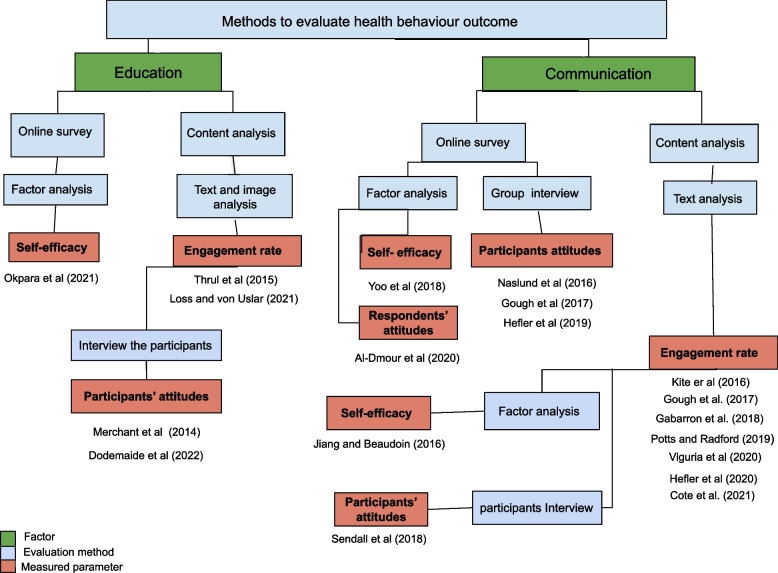
study design used in the studies to evaluate the outcome

The concept map (Fig. 5) structured the methods used in the selected research papers based on the functionality of social media to evaluate the outcome of health promotion campaigns for behaviour change. The review identified communication and education as the most influential factors to evaluate the outcome of health promotion programs for behaviour change [27].

### Communication factor to evaluate the sustainability of health promotion outcome

Of the nine studies that adopted communication factor to evaluate the outcome of the health promotion program [5, 12, 29–32, 35, 37, 38], seven conducted content analysis (text analysis) to measure the engagement rate [29–31, 35, 37], exposure/control rate [38] and self-efficacy [32] as the parameters to evaluate the outcome. Three studies implemented online surveys [5, 12, 30] and used factor analysis [5, 12] and focus group interviews [30] to measure self-efficacy [12], respondents’ attitude [5] and participants’ attitude and evaluate the outcome.

### Content analysis to evaluate communication outcome

A national mental health campaign involved a content analysis of suicide-related posts on Twitter associated with the campaign to determine the association between change in the number of suicides during and immediately following the campaign [38]. Counts during exposure and control windows were compared. The content of tweets related to eating disorder campaigns were analysed according to five categories of promotion health behaviour stages in eating disorders and measured the parameters of potential impact, potential reach, number of contributors, percentage of very active users, and percentage of very influential users to evaluate the outcome [37]. The reactions of Twitter users were measured in an oral health campaign by performing content analysis of the tweets and manually classifying the tweets based on Neiger’s model of social media engagement [35, 45]. One study conducted a content analysis of the China Tobacco Control Media Campaign on Sina Weibo and dummy coded the segmented posts to three persuasive content characteristics for behaviour change strategy [32]. They used regression to test the effects of the independent variables on online audience engagement. The Twitter account of a regional cancer charity who had hosted a prevention skin cancer campaign were evaluated [30]. The volume of tweets related to a list of predefined keyword search terms was compared pre and post campaign to track the messages based on the location of the participants. They measured the most common social media metrics such as impressions, engagements, engagement/impression rate, likes and shares to show when a message spreads by detailing the number of users who see it, who respond to it, or who subsequently share that message with their friends or followers.

### Online surveys to evaluate communication outcome

One researcher evaluated the outcome of a cervical cancer prevention campaign by conducting an online survey engaging the social media users who followed the campaign [12]. One study investigated the health behaviour of social media users on 3 social media channels (Facebook, Twitter, and Instagram) and the online discussions concerning diabetes [29]. They surveyed the users of diabetes social media groups through an online questionnaire to assess the impact of health promotion intervention on their lifestyles and online health behaviours [29]. A quasi-experimental study assessed the feasibility of social media intervention by conducting a web-based survey that was promoted on social media platforms [30]. One study developed an integrated conceptual model and assumed social media interventions would increase public protection and prevention through the interaction between public awareness and behaviour changes as mediating factors. They conducted an online survey questionnaire to test the defined hypothesis [5].

### Education factor to evaluate the sustainability of health promotion outcome

From four studies adopting the education factor [10, 33, 34, 36], three studies used content analysis (text and image) to measure the engagement rate [33, 36] and attitude of the interviewed participants [34]. One study conducted online surveys to measure self-efficacy [10] to evaluate the outcome of health promotion campaigns for behavioural change.

### Content analysis to evaluate education outcome

One researcher performed content analysis of text and image data on the Facebook account of public and private health insurance providers related to health topics [33]. One study conducted a Facebook campaign to actively target young adults applying the stage of behaviour change of TTM by measuring the engagement of users in sharing comments on Facebook instead of likes [36]. They used t-tests and negative binomial regression analysis using TTM to examine the relationship between engagement and characteristics of participants. The TTM based post content was dummy coded and the deviation was used to estimate the difference in engagement between each individual post and the overall engagement [36]. One study conducted an educational intervention campaign with 5 self-regulatory techniques. The participants were encouraged to engage in an existing social network to meet their weight loss achievements. The level of engagement with the Facebook post was quantified and a subset of participants were interviewed to evaluate the passive online engagements [34].

### Online survey to evaluate education outcome

An online survey was conducted to explore the moderating role of colour in YouTube animated cartoons on health behaviour of social media users [10]. A hierarchical multiple regression model was used to compute the capacity of YouTube animated cartoons regarding Covid-19 in predicting the health behaviour of social media users. The results show that colour significantly moderate the impact of COVID-19 YouTube animated cartoons on health behaviours such as avoiding handshake, regular hand washing, using hand sanitizers and face masks. They used self-efficacy to evaluate the outcome of sustainable health behaviour among social media users who were exposed to COVID-19 YouTube animated cartoons [10].

## Discussion

Evidence shows that the awareness initiatives could increase the rate of reach, exposure, impression, impact and engagement of social media users in health promotion programs and impact on health knowledge and behaviour outcomes [5]. Most research addresses specific interventions and approaches, which vary widely in focus, target population, theoretical foundations, mode of delivery, functionality, and usability. This variation makes it difficult to find out what works and how, and it complicates efforts to compare approaches [27].

Recent reviews of social media interventions for health behaviour change have concluded that these kinds of interventions can have small but significant beneficial effects on health behaviour change. The low participant engagement with social media interventions is a critical obstacle to improving health behaviour outcomes [36]. Some studies focused on analysis of the content of the posts on social media in raising awareness rather than promoting treatment, help-seeking behaviours and resilience, which would likely have a more significant behaviour change outcome [3, 37]. Other studies found social media as a tool to engage participants in the health promotion programs and other health behaviour change interventions, although no participant baseline characteristics significantly predicted the engagement in the studies [36]. Another study suggested that use of social media platforms can positively influence awareness of public health, behavioural changes and public health protection [5].

The effectiveness of social media in public health and medicine are seen in the systematic review literature but definitive conclusions cannot be made at this time. However, the systematic review reported harms on all groups include the impact of social media on mental health, privacy, confidentiality and information reliability [1]. Some studies revealed that Twitter [35, 37, 38] and Facebook [36] could not promote any specific preventive or help-seeking behaviour changes although the highest impact and most influential contribution were observed, and the participants demonstrated a statistically significant improvement in health literacy and a higher willingness to access additional information.

Behavioural change theories can help public health authorities and social media initiatives to understand the process of changing in health behaviours and enabling them to modify health promotion interventions [5]. Theory-driven interventions are effective tools that can promote sustainable and positive health behaviour changes in real-time [26]. One study suggested that self-efficacy impacts on users’ communicative behaviours on social media channels which can ultimately affect the users’ health behaviours [12].

Despite the growth of literature examining social media in the health context, limited studies provided insight into how the application of social media may vary in various public health interventions [5]. Existing research employed different frameworks to understand how to select the most relevant health behaviour, how to apply the most appropriate methods to evaluate the outcomes and how to effectively harness social media uses in health promotion interventions [18]. Analysing the users’ engagement with online health information and monitoring the trend of users’ behaviours on social media can be used to improve the usage of social media to change health behaviours in the future health promotion campaigns [29]. Whether health communication on social media can actually lead to behaviour changes still needs to be evaluated [33].

However, it is important to acknowledge that engagement with social media does not necessarily reflect real-life behaviour [35] and simply measuring the metrics related to the activity of users on social media does not necessarily demonstrate the behaviours of the social media users [46]. No consensus has emerged yet on whether this medium has the potential to facilitate or undermine public health efforts and ultimately promote health behaviours [5, 18]. There is no widely accepted conceptual model exists for examining the roles that social media can play with respect to promotion of public health [18].

## Conclusion

Evaluating the performance of social media campaigns to promote health behaviours towards establishing a healthy lifestyle is a complex process. Emerging research focused on evaluating the potential of social media as an opportunity to create awareness. The examples of functions that can influence the outcomes relevant to the evaluation of the effects of social media on the public health are minimising the spread of misinformation and increasing public awareness of accurate health-related information, enhancing real-time surveillance related to incident disease, disease Control and mitigation, and screening or treatment interventions. Further research attempted to go beyond awareness, and employ social media to evaluate the success of health promotion campaigns for a range of health behaviour and prevention topics including controlling overweight and obesity, smoking cessation, cancer prevention, control diabetes, promotion of oral health, eating disorder prevention, COVID-19 prevention, suicide prevention and mental health and wellbeing.

The review identified that most studies adopted quantitative methods to evaluate the health behaviour outcomes on social media. Facebook and YouTube were effectively used for intervention and education purposes to change health behaviour and Twitter and Instagram were used more to observe the trend of changes in health behaviour. Majority of studies adopted Social Cognitive Theory (SCT) as the health promotion framework to evaluate health behaviour change. Most papers used social media for communication rather than education to evaluate the outcome for behaviour change.

However, social media interactions provide rich data to understand the processes of stages of behaviour change and can help to discover the patterns of changes in health behaviour in a target population. Health promotion programs using social media platforms require the incorporation of some theories of behavioural change in social media interventions in order to pursue the shifts in stages of behaviour change to evaluate the sustainability of health behaviour outcomes. Health promoters need to strategically design their campaigns so that their messages further lead to actual health behaviour being promoted and also to attend to the tractable nature of social media, which may further facilitate subjective norms among users.

## Data Availability

All data analysed during this study are included in this published article.

## Abbreviations

TTM: Transtheoretical Model
SCT: Social Cognitive Theory
LM: Laugh Model
Covid-19: Coronavirus disease of 2019
